# A Phantom of the Large Bowel

**DOI:** 10.7759/cureus.5738

**Published:** 2019-09-24

**Authors:** Shivantha Amarnath, Fady G. Haddad, Deeb Liliane

**Affiliations:** 1 Internal Medicine, Staten Island University Hospital, Northwell Health, Staten Island, USA; 2 Gatsroenterology, Staten Island University Hospital, Northwell Health, Staten Island, USA

**Keywords:** giant colonic diverticulum, diverticulosis, diverticulitis, sigmoid colon

## Abstract

Giant colonic diverticulum (GCD) is a diverticulum that is larger than 4 cm in diameter most commonly arising from the sigmoid colon. Patients with GCD are typically asymptomatic, and clinical manifestations vary from a soft palpable abdominal mass to diarrhea, which is well described in the literature. However, few studies have demonstrated a presentation of GCD with acute diverticulitis. Herein, we report a case of a middle-aged patient presenting with sepsis due to acute diverticulitis as an initial presentation of a GCD.

## Introduction

Giant colonic diverticulum (GCD), commonly referred to as giant gas cyst or giant colon cyst, is a rare entity that was first described in 1946 [1]. It is defined as a colonic diverticulum that is larger than 4 cm in size, which could be an isolated finding commonly in the sigmoid colon or associated with concomitant diverticular disease in 85% of the cases. Patients are typically asymptomatic. However, clinical manifestations vary from lower abdominal pain, constipation, rectal bleeding, to a soft palpable abdominal mass. Unusually, a carcinoma may develop in the diverticular mucosa or patients could acutely present with a constellation of symptoms denoting acute diverticulitis. Management usually consists of an en-bloc resection of the affected colon segment with primary colonic anastomosis. To our knowledge, our patient is one of the very few cases reported in the literature to present as such. He is a middle-aged man who complained of non-specific abdominal pain and was diagnosed with acute diverticulitis of a GCD.

## Case presentation

**Figure 1:**
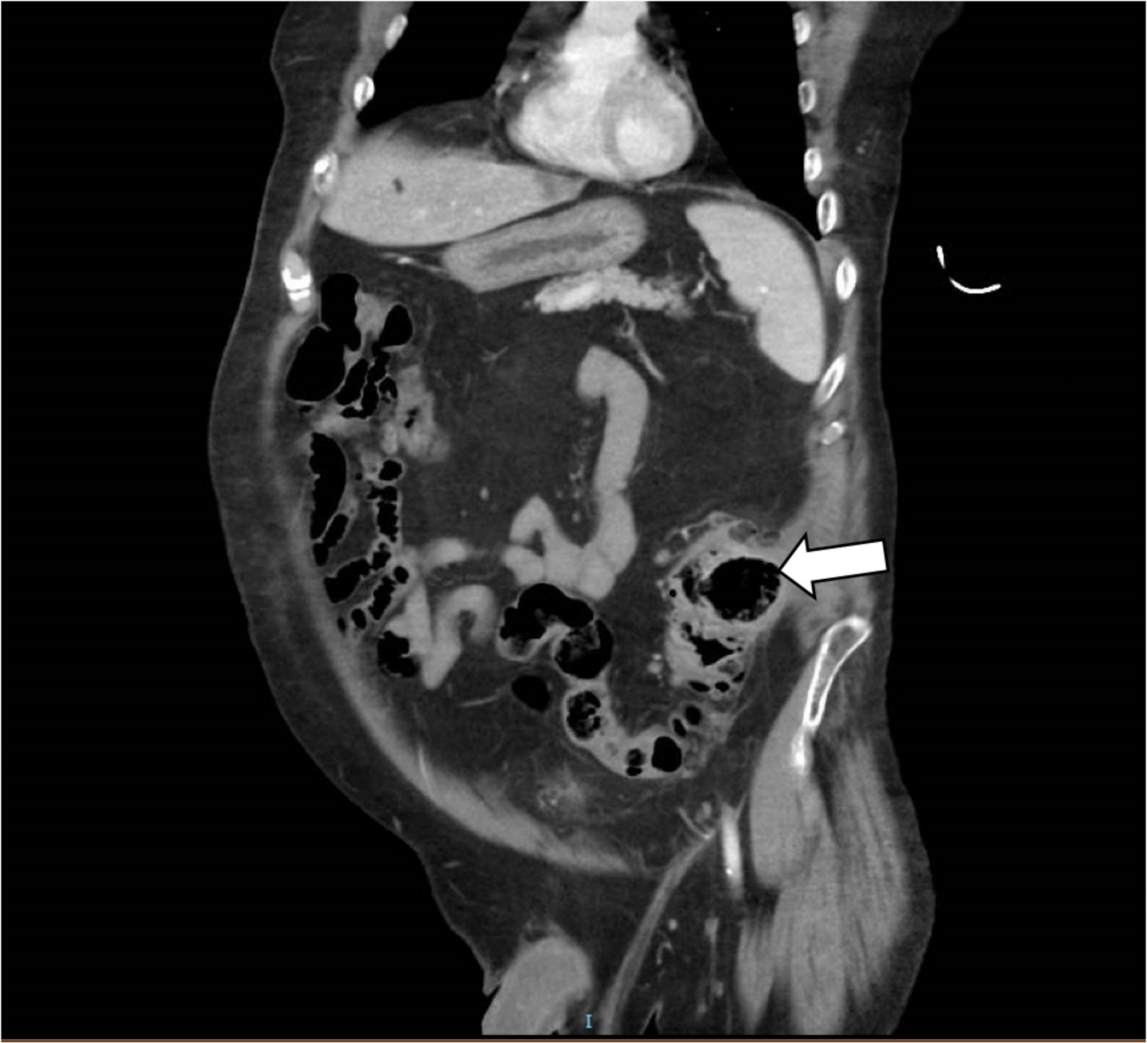
Coronal view of an enhanced abdominal CT scan showing a large air-filled thin-walled cavity arising from the sigmoid colon (arrow)

**Figure 2:**
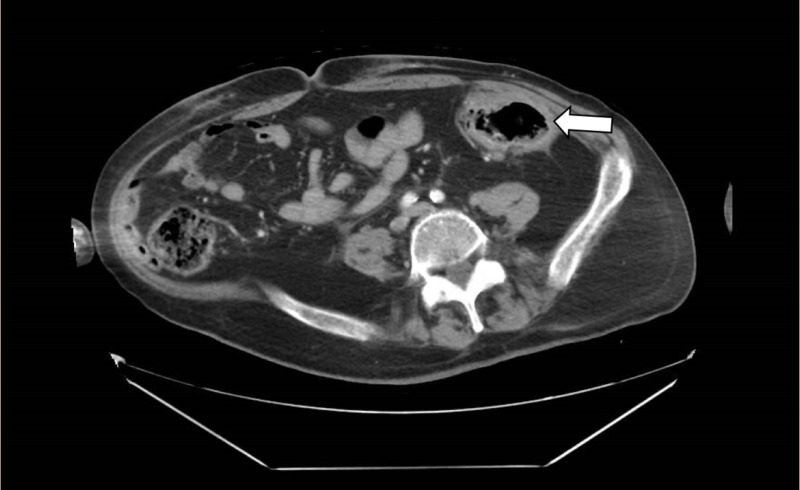
Axial view of an enhanced abdominal CT scan showing a large thin-walled cyst with air-fluid levels arising from the sigmoid colon (arrow)

A 63-year-old male with a history of multiple sclerosis and major depression presented to our hospital with generalized abdominal pain of one-day duration. There were no other associated symptoms. Vital signs were within normal limits except for sinus tachycardia, and fever of 101°F. Physical examination was remarkable for lower abdominal tenderness with no rigidity or guarding. Laboratory studies revealed leukocytosis of 23,400/mm^3^ (normal range: 4,800/mm^3^-10,800/mm^3^) with a neutrophilic predominance and lactic acid of 3 mmol/L (normal range: 0.5-2.2 mmol/L). The patient underwent a contrast-enhanced computed tomography (CT) scan of the abdomen and pelvis revealing an isolated 4.5 cm diverticulum at the sigmoid colon with surrounding inflammatory changes consistent with acute diverticulitis (Figures 1, 2). There was no evidence of a focal drainable abscess or free air. The patient was admitted for sepsis due to acute diverticulitis of the GCD and was started on systemic antibiotics. He improved clinically, tolerated oral diet, and was successfully discharged three days after presentation.

## Discussion

A GCD is a rare complication of diverticular disease [2]. It was first described in 1946 by two French physicians, Bonvin and Bonte, who defined this condition as a diverticulum that is larger than 4 cm in diameter [2, 3]. Typically, diverticula do not enlarge greater than 1 to 2 cm in size; however, in the case of a GCD, size may vary over time, reaching up to 40 cm [3]. In such instances, a soft abdominal mass may be palpated; hence, this condition is occasionally referred to as a “phantom tumor” [3]. GCD typically arises from the sigmoid colon, and rarely from the transverse colon, but it is reported to occur in any segment of the colon [2]. In general, this entity affects the elderly population with an age range of 40 to 90 years and mostly occurring after the age of 60 [4].

The pathogenesis of GCD is not clearly understood. Two theories have been proposed. A ball and valve mechanism involving communication between the bowel lumen and diverticulum where a one-way valve permits the entry of air into the diverticulum, thus progressively raising its intraluminal pressure and causing persistent enlargement of the diverticulum [3]. An alternate theory is that gas-forming bacteria encased within a cyst of the colon after the neck becomes obliterated due to chronic inflammation will gradually distend and increase the size of the diverticulum. These two principal theories may practically co-exist to give rise to a GCD [3].

Histologically, GCD can be classified into three types [4, 5]. Type 1 GCD is a small pulsion diverticulum where the true muscularis propria layer ends at the neck of the diverticulum and remnants of the muscularis mucosa with granulation tissue are found within the wall of the diverticulum [4, 6]. Type 2 GCD, which is the most commonly encountered, refers to an inflammatory GCD that was formed due to a perforation in the subserosal layer leading to a walled-off abscess cavity contributing a gradually enlarging diverticulum [6]. The least common type 3 GCD denotes a true diverticulum comprising all layers of the colonic wall. The latter mostly encountered in the pediatric population and is often considered as a congenital diverticulum [6, 7].

The entity is usually asymptomatic, but clinical manifestations are variable and non-specific when they occur. They include abdominal pain, constipation, the sensation of an abdominal mass, vomiting, diarrhea, and rarely, bleeding per rectum [6]. Urinary symptoms and transient leg swelling due to compression of the bladder and iliac veins respectively, as well as focal neurological deficits, are some of the unique presentations encountered in this condition [1, 8]. Differential diagnosis may include and not limited to Meckel’s diverticulum, small bowel duplication cyst, emphysematous cholecystitis, pancreatic pseudocyst, and vesicoenteric fistula [4]. There have been case reports of malignant tumor growth in the diverticula, namely colonic adenocarcinoma and MALT (mucosa-associated lymphoid tissue) lymphoma [9, 10]. Complication-related mortality often ranges from 10 to 40% [11].

Imaging studies usually suggest the diagnosis: abdominal X-ray may demonstrate a balloon sign in most cases, which appears as a sizeable gas-filled cyst that is either round or oval in appearance [1, 3]. The most definitive test is a CT scan of the abdomen and pelvis demonstrating a smooth-walled cavity in part of the affected colon with an air-fluid level. The wall may contain calcifications from chronic inflammation. A thickened wall that enhances with contrast is associated with acute inflammation, correlating with diverticulitis. With the widespread use of CT scan in recent years, there has been a diminution of the utility of barium enemas in the diagnosis of GCD as it is associated with a high risk of perforation. Colonoscopy also carries a slightly increased risk and should be avoided whenever possible.

The preferred treatment of an uncomplicated GCD consists of an en-bloc resection of the affected colon segment with primary colonic anastomosis [2]. In hemodynamically compromised patients, an emergent Hartmann’s resection might be performed, followed by a second-stage surgical procedure to restore the intestinal continuity [3]. However, it should be noted that in high-risk surgical candidates with acute inflammation of GCD, a more conservative approach should be taken at initial presentation, and this ranges from parenteral antibiotics, like in our patient, to percutaneous drainage of any superimposed abscesses [6, 12].

## Conclusions

GCD is a rare complication of colonic diverticulosis. Clinical presentation is very non-specific with variable signs and symptoms and requires the use of imaging modalities to delineate the diagnosis. The gold standard of treatment in symptomatic GCD consists of surgical segment resection of the affected colon with primary anastomosis. Conservative measures should be preserved to individuals who are either asymptomatic or deemed to be at high surgical risk.
